# Computed tomography Hounsfield units can predict breast cancer metastasis to axillary lymph nodes

**DOI:** 10.1186/1471-2407-14-730

**Published:** 2014-09-30

**Authors:** Masakazu Urata, Yuko Kijima, Munetsugu Hirata, Yoshiaki Shinden, Hideo Arima, Akihiro Nakajo, Chihaya Koriyama, Takaaki Arigami, Yoshikazu Uenosono, Hiroshi Okumura, Kosei Maemura, Sumiya Ishigami, Heiji Yoshinaka, Shoji Natsugoe

**Affiliations:** Department of Digestive Surgery, Breast and Thyroid Surgery, Graduate School of Medical and Dental Sciences, Kagoshima University, 8-35-1, Sakuragaoka, Kagoshima, 890-8520 Japan; Department of Epidemiology and Preventive Medicine, Graduate School of Medical and Dental Sciences, Kagoshima University, Kagoshima, Japan

**Keywords:** Breast cancer, Computed tomography, Hounsfield unit, Lymph node metastasis, Diagnosis, Axillary lymph node

## Abstract

**Background:**

Axillary lymph node (ALN) status is an important prognostic factor for breast cancer. We retrospectively used contrast-enhanced computed tomography (CE-CT) to evaluate the presence of ALN, metastasis based on size, shape, and contrasting effects.

**Methods:**

Of 131 consecutive patients who underwent CE-CT followed by surgery for breast cancer between 2005 and 2012 in our institution, 49 were histologically diagnosed with lymph node metastasis. Maximum Hounsfield units (HU) and mean HU were measured in non-contrasting CT (NC-CT) and CE-CT of ALNs.

**Results:**

Of 12 examined measurements, we found significant differences between negative and metastatic ALNs in mean and maximum NC-CT HU, and mean and maximum CE-CT HU (*P* < 0.05). We used a receiver operating curve, to determine cut-off values of four items in which significant differences were observed. The highest accuracy rate was noted for the cut-off value of 54 as maximum NC-CT HU for which sensitivity, specificity, and accuracy rate were 79.6%, 80.5% and 80.2%, respectively.

**Conclusions:**

CT HU of a patient with breast cancer are absolute values that offer objective disease management data that are not influenced by the screener’s ability.

## Background

Breast cancer is the most common newly diagnosed cancer type and the fifth most common cause of cancer-related death among women in Japan [1]. Status of axillary lymph nodes (ALNs) is the most important predictor of survival [2].

Manual palpation is a well-known method that is used to non-invasively detect ALN metastasis [3]; however, it has low specificity and sensitivity and cannot accurately predict the ALN status [4]. Contrast-enhanced computed tomography (CE-CT) has been used to evaluate ALN metastasis based on size, shape, and contrasting effects [5–10]. Use of whole-body ^18^ F-fluorodeoxy glucose- positron emission tomography (FDG-PET)/CT for breast cancer staging and treatment monitoring has also recently increased due to its ability to detect previously unknown metastases [11–16]. However, its diagnostic accuracy for ALN staging has not yet been established [16, 17].

Hounsfield units (HU) are a measure of X-ray attenuation in CT images. This retrospective study evaluated ALN metastasis against HU to determine optimal cut-off values.

## Methods

### Patients

Between January 2005 and May 2012, 283 consecutive patients with invasive ductal carcinoma (IDC) underwent breast and axillary surgery in our hospital without any neoadjuvant systemic therapy such as chemotherapy or endocrine therapy. Of these patients, 131 underwent CE-CT prior to surgery in our hospital using the same system, and were enrolled in this study; the other 152 patients underwent CE-CT using a different system in another hospital. All subjects had undergone mammography, ultrasonography (US), core needle biopsies for their primary breast lesions, bone scintigraphy for preoperative staging. Magnetic resonance imaging, FDG-PET, and pathological or cytological examinations for ALNs, depending on the case. Sentinel lymph node (SLN) biopsies were performed on 51 patients with diagnoses of clinical T1N0M0 breast cancer according to the TNM classification [18]. The dye and radioisotope method used to detect SLNs was reported previously [19]. Axillary lymphadenectomy was performed on 27 and 18 patients who were diagnosed with T1-4N1M0 or T2-4N0M0, respectively. Of 51 patients who underwent SLN biopsies, 9 received additional axillary lymphadenectomy due to findings of metastasis by the intraoperative histological examination.

Patient characteristics and the pathological and surgical findings were collected from our database records and individual patient electronic medical records, and was approved by the Institutional Review Board of the Kagoshima University Medical and Dental Hospital. We received informed consent from each study participant and approval for our protocol from the Ethics Committee of Kagoshima University.

Table 1 shows clinicopathological features of the 131 patients enrolled in this study. Their average age was 58.3 years (range: 21–95 years). One patient was diagnosed with T1mic cancer, 73 with T1, 32 with T2, 7 with T3, and 18 with T4 lesions. Total and partial mastectomies were performed on 74 and 57 patients, respectively. SLN biopsy was performed on 42 patients, SLN biopsy followed by axillary lymphadenectomy on 9 patients, and axillary lymphadenectomy on 80 patients. Of the 131 patients, 49 had lymph node metastasis. All metastases were evaluated as macrometastasis (Table 1).

**Table 1:** **Clinical characteristics of the 131 patients**

Variable	
Age in years, average (range)	58.3 (21–95)
Menopause	
Pre	41 (31.3%)
Post	90 (68.7%)
Pathological T stage	
T1mic	1 (0.8%)
T1a	11 (8.4%)
T1b	21 (16.0%)
T1c	41 (31.3%)
T2	32 (24.5%)
T3	7 (5.3%)
T4	18 (13.7%)
Procedure	
Total mastectomy	74 (56.5%)
Partial mastectomy	57 (43.5%)
Lymph node dissection	
SNB* without axillary lymphadenectomy	42 (32.1%)
SNB* with axillary lymphadenectomy	9 (6.9%)
Axillary lymphadenectomy	80 (61.0%)
Lymph node metastasis	
Positive	49 (37.4%)
Negative	82 (62.6%)

*Sentinel lymph node biopsy.

### Testing set

All 95 consecutive patients with IDC who underwent breast and axillary surgery in our hospital between June 2012 and July 2014 with no neoadjuvant therapy underwent CE-CT: 40 before surgery in our hospital using the same system, and the other 55 patients using a different system in another hospital. We enrolled the 40 former patients in this testing set.

### CT scanning

**Figure 1:**
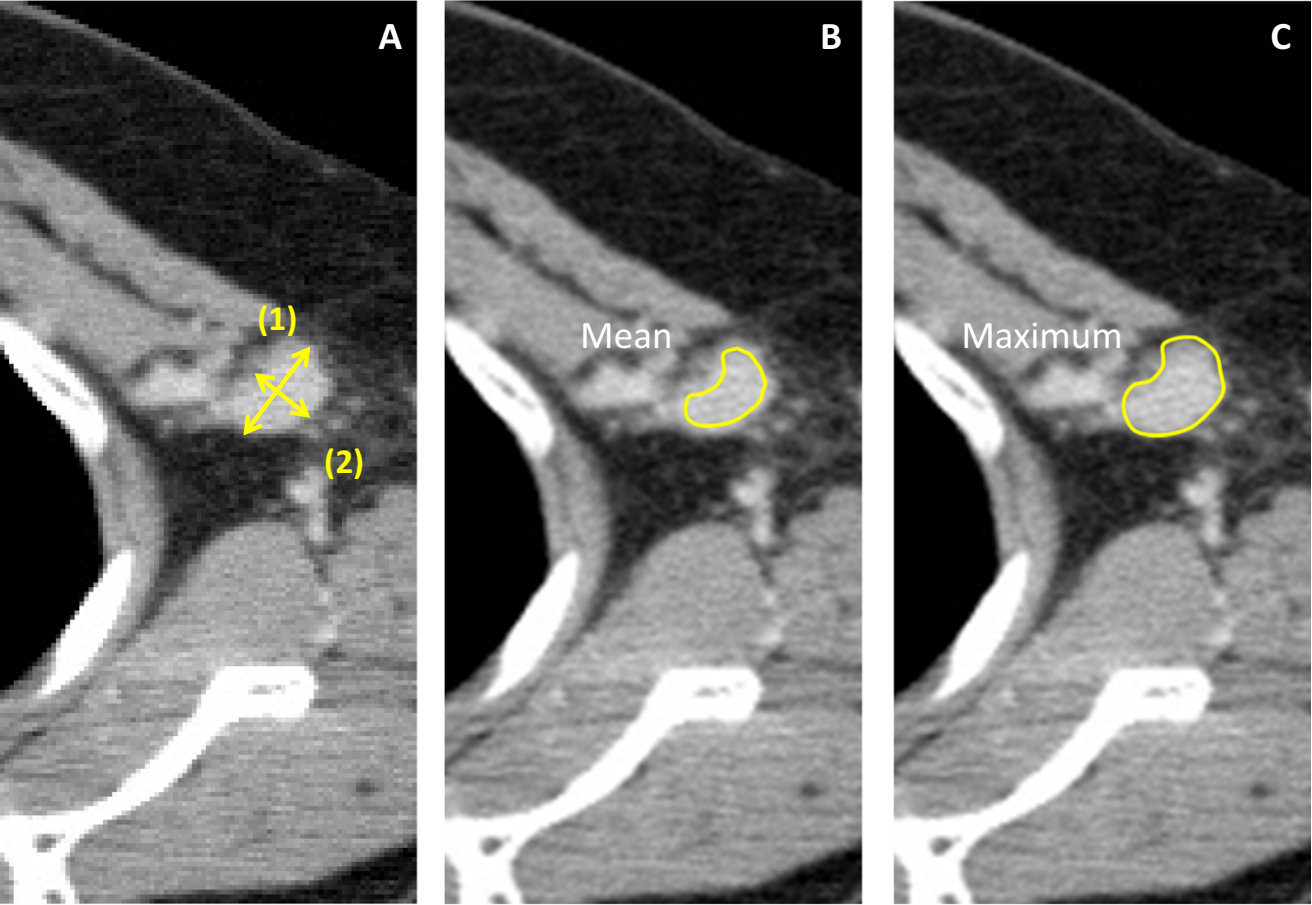
**Measurement of axillary lymph nodes in a patient with invasive ductal carcinoma in the left breast. A**. ALN sizes were measured in the maximum sectioned surface of the CT image with 3-mm slices: (1) Longest transverse diameter of the oval lymph node; (2) Shortest transverse diameter of the oval lymph node; **B**. To measure mean HU, we selected a range of interest (ROI) such that the line did not protrude from the ALN edge. **C**. ROI surrounded the lymph node, and the maximum HU inside of the ROI was measured. We took extra care not to include bone or blood vessels near the ROI.

Patients were examined in a supine position with their arms stretched above their heads at the end of inspiration using a CT scanner (Aquilion TM64, Toshiba Medical, Tokyo, Japan). Scanning parameters included 120 kVp, a 0.5-second tube rotation, 53° helical pitch, 206-mm table speed, 0.5 second gantry rotation time, and 3-mm thick reconstructed sections. Images were analyzed on SYNAPSE (Fuji Film, Inc., Tokyo, Japan).We observed 437 ALNs in 131 patients on CT. On some slides, in which both the pectoralis major and minor muscles were detected, we evaluated all lymph nodes without any information on pathological findings. The mean number of evaluated lymph nodes in each patient was 3.34 (range: 2–6). Maximum and mean HU were measured in non-contrasting CT (NC-CT) and CE-CT ALNs, contralateral ALNs, the aortic inside arch and the pectoralis major muscle. To measure lymph node HU with/without internal fat at normal lymph node hila, we traced outlines of lymph nodes by hand to highlight a range of interest (ROI) (Figure 1). To exclude artificial contamination, we excluded these outlines when we evaluated mean HU values, but included them to determine maximum HU values. On each lymph node, we also evaluated long- and short-axis diameters, and internal fat density, which indicated absence of central images for bilateral ALNs. The highest value of a patient’s several lymph nodes was used as the value for that patient. Mean HU, maximum HU, and primary tumor size were simultaneously determined in the same manner.

### Pathological evaluation

All dissected ALNs were cut into single sections and stained with hematoxylin–eosin for analysis by a pathologist. Both micro- and macro-metastasis were considered positive.

### Statistical analysis

**Figure 2:**
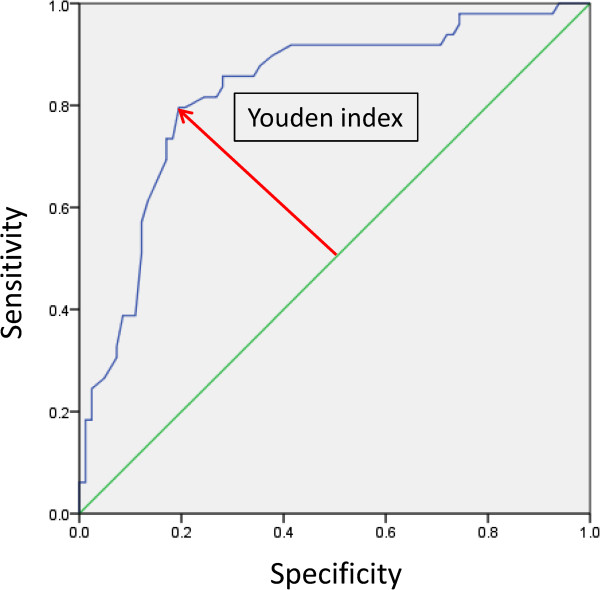
**ROC curve of maximum HU of an unenhanced lymph node CT.** The AUC of this case was 0.827. We made a ROC curve and found the cut-off value using the Youden index.

Data were evaluated using SPSS Ver.20 software (IBM SPSS, Chicago, IL). Student’s *t*-test was used to assess differences between the groups. *P* < 0.05 was considered significant. We examined mean HU and maximum HU of the ALNs and primary tumor on both NC-CT and CE-CT. We examined differences between metastatic lymph nodes and non-metastatic lymph nodes using 18 measurements, as shown in Table 2. We then used the Youden index of receiver operating characteristic (ROC) curve to determine cut-off values for items in which a significant difference was observed in the *t*-test. The ROC curve was drawn using SPSS Ver.20 software (Figure 2, for an example).

**Table 2:** **Comparisons between negative and positive lymph nodes for 12 CT measurements**

	Lymph node metastasis		
Factors (mean ± SD)	Negative (***n*** = 82)	Positive (***n*** = 49)	***P***
Mean NC-CT HU of lymph node	7.83 ± 22.31	29.17 ± 16.37	< 0.05
Maximum NC-CT HU of lymph node	34.28 ± 23.58	60.06 ± 17.52	< 0.05
Mean CE-CT HU of lymph node	52.45 ± 27.29	78.09 ± 22.49	< 0.05
Maximum CE-CT HU of lymph node	86.95 ± 27.58	118.63 ± 23.14	< 0.05
Mean NC-CT HU of primary tumor	36.09 ± 18.88	37.28 ± 16.58	0.89
Maximum NC-CT HU of primary tumor	69.30 ± 30.65	78.17 ± 18.55	0.11
Mean CE-CT HU of primary tumor	91.92 ± 28.36	89.03 ± 31.45	0.39
Maximum CE-CT HU of primary tumor	135.81 ± 31.27	148.88 ± 25.32	0.88
Long-axis diameter of lymph node (mm)	6.05 ± 2.32	10.37 ± 4.75	< 0.05
Short-axis diameter of lymph node (mm)	4.42 ± 1.71	7.35 ± 3.39	< 0.05
Long-axis diameter of primary tumor (mm)	18.71 ± 10.14	40.54 ± 37.94	< 0.05
Short-axis diameter of primary tumor (mm)	12.99 ± 7.11	25.44 ± 33.37	< 0.05

CE-CT: contrast-enhanced computed tomography; HU: Hounsfield unit; NC-CT: non-contrasting computed tomography; SD: standard deviation.

## Results

### HU value and lymph node metastasis

Mean NC-CT HU was 7.83 ± 22.31 for non-metastatic lymph nodes and 29.17 ± 16.37 for positive nodes. Similarly, maximum NC-CT HU, mean CE-CT HU, and maximum CE-CT HU were 34.28 ± 23.58, 60.06 ± 17.52 and 52.45 ± 27.29 for negative nodes; and 78.09 ± 22.49, 86.95 ± 27.58 and 118.63 ± 23.14 for positive nodes, respectively (Table 2). We also analyzed associations between pathological findings for metastasis and NC-CT and CE-CT HU values for ALNs, primary tumors, and long- and short-axis diameters (Table 2).

Of the 12 values, mean ALN NC-CT HU, maximum ALN NC-CT HU, mean ALN CE-CT HU, maximum ALN CE-CT HU, long- and short-axis ALN diameters, and long- and short-axis diameters of the primary tumor differed significantly for metastatic and negative ALNs; with maximum and mean HU significantly higher in positive nodes. However, in the primary tumors, none of the CT values correlated with ALN metastasis (Table 2).

### Lymph node metastasis and diameters of the lymph node and primary tumor

Long- and short-axis diameters of metastatic lymph nodes were significantly larger than for negative nodes (*P* < 0.05, Table 2). Long- and short-axis diameters of primary tumors correlated with lymph node metastasis (*P* < 0.05 for both).

### Diagnostic accuracy of HU values in detecting lymph node metastasis

For the four items with significantly higher HU values in metastatic lymph nodes—mean and maximum NC-CT HU values, and mean and maximum CE-CT HU values (Table 3)—we determined HU cut-off values, using the Youden index for ROC curves (Figure 2). The highest accuracy rate was found for maximum ALN NC-CT HU at a cut-off value of 54 (sensitivity: 79.6%; specificity: 80.5%; positive predictive value [PPV]: 70.9%; negative predictive value [NPV]: 86.8%; accuracy: 80.2%), followed by maximum ALN CE-CT HU at a cut-off value of 103 (sensitivity: 83.7%; specificity: 72.0%; PPV: 64.1%; NPV: 88.1%; accuracy: 76.3%), and mean ALN CE-CT HU at a cut-off value of 16 (sensitivity: 83.7%; specificity: 64.6%; PPV: 58.6%; NPV: 86.9%; accuracy: 71.8%). In the same way, we determined size cut-off values and listed sensitivity,specificity, PPV, NPV, and accuracy (Table 4).

**Table 3:** **HU measurements that differed significantly in metastatic and negative lymph nodes**

	Cutoff value	Sensitivity	Specificity	PPV	NPV	Accuracy
	(HU)	(%)	(%)	(%)	(%)	(%)
Lymph node NC-CT						
Mean HU	16	83.7	64.6	58.6	86.9	71.8
Maximum	54	79.6	80.5	70.9	86.8	80.2
Lymph node CE-CT						
Mean HU	60	85.7	58.5	55.3	87.3	68.7
Maximum HU	103	83.7	72	64.1	88.1	76.3

CE-CT: contrast-enhanced computed tomography; HU: Hounsfield unit; NC-CT: non-contrasting computed tomography; NPV: negative predictive value; PPV: positive predictive value.

**Table 4:** **Size measurements that differed significantly in metastatic and negative lymph nodes**

	Cut-off value	Sensitivity	Specificity	PPV	NPV	Accuracy
	(mm)	(%)	(%)	(%)	(%)	(%)
Lymph node						
Long-axis dia	7.42	75.5	74.4	63.8	83.6	74.8
Short-axis dia	4.96	81.6	73.2	64.5	87	76.3
Primary tumor						
Long-axis dia	17.08	91.7	56.1	91.7	56.1	69.2
Short-axis dia	16.27	57.1	81.7	65.1	76.1	72.5

Dia: diameter; NPV: negative predictive value; PPV: positive predictive value.

### Evaluations of testing set

We used cut-off values for mean and maximum HU values of both NC-CT and CE-CT which were derived from 131 cases, to evaluate several lymph nodes in one patient, which we compared with pathologists’ findings, as shown in Table 5.

**Table 5:** **Testing set results for lymph node metastases; CT values compared with pathological findings**

Mean NC-CT HU		Pathological findings		
		Positive	Negative	Total
CT^†^	Positive	9	9	18
	Negative	1	21	22
	Total	10	30	40
Sensitivity 90.0%, Specificity 70.0%, PPV 50.0%, NPV 95.5%, Accuracy 75.0%				
**Maximum NC-CT HU**		**Pathological findings**		
		**Positive**	**Negative**	**Total**
CT^†^	Positive	10	7	17
	Negative	0	23	23
	Total	10	30	40
Sensitivity 100.0%, Specificity 76.7%, PPV 58.8%, NPV 100.0%, Accuracy 82.5%				
**Mean CE-CT HU**		**Pathological findings**		
		**Positive**	**Negative**	**Total**
CT^†^	Positive	7	13	20
	Negative	3	17	20
	Total	10	30	40
Sensitivity 70.0%, Specificity 56.7%, PPV 35.0%, NPV 85.0%, Accuracy 60.0%				
**Maximum CE-CT HU**		**Pathological findings**		
		**Positive**	**Negative**	**Total**
CT^†^	Positive	8	12	20
	Negative	2	18	20
	Total	10	30	40
Sensitivity 80.0%, Specificity 60.0%, PPV 40.0%, NPV 90.0%, Accuracy 65.0%				

^†^Diagnosis of metastasis through CT HU values.

CE-CT: contrast-enhanced computed tomography; HU: Hounsfield unit; NC-CT: non-contrasting computed tomography; NPV: negative predictive value; PPV: positive predictive value.

## Discussion

The 10-year survival rate of patients with ALN metastasis depends on the number of involved nodes, and ranges from 30% for those with > 10 metastases to 90% for those with no metastasis [2]. The ALN status is not only important for estimating prognoses, but also for selecting individual treatment regimens [20]. The American College of Surgeons Oncology Group (ACOSOG) Z0011 trial showed that patients randomized to SLN dissection (SLND) alone or to SLND + ALN dissection (ALND), did not significantly differ in local or regional recurrence [21]. The ACOSOGZ0011 enrolled patients who were diagnosed as N0 before randomization, which supports the importance of preoperative ALN evaluation. Our present study could facilitate these evaluations.

The use of CT to assess ALN metastasis has been reported previously [5–10]. Relatively good results were reported for various criteria used to detect lymph node metastasis, such as short-axis ALN diameter, ratio of long- to short-axis ALN diameters, enhancement type, shape, or intra-nodal fat density [10, 22–24]. Such methods are useful for detecting ALN metastasis, but may strongly depend on the personal ability of the screener.

Use of US in diagnosing ALN metastasis is also widely reported [25, 26]. Results of US for non-palpable axillary nodes based on nodal size showed that sensitivity varied between 48.8% (95% confidence interval: 39.6–58%) and 87.1% (76.1–94.3%) and specificity varied between 55.6% (44.7–66.3%) and 97.3% (86.1–99.9%); for lymph node morphology, sensitivity ranged from 26.4% (15.3–40.3%) to 75.9% (56.4–89.7%) and specificity ranged from 88.4% (82.1–93.1%) to 98.1% (90.1–99.9%) [25]. We previously reported that US screening of ALNs was useful both for diagnosing nodal metastasis, and for predicting prognoses. We showed that US sensitivity, specificity and accuracy rates were 69.5%, 85.8% and 79.7%, respectively [26]; however, its ease of use is somewhat offset by the influence of operator’s skill and possible subjectivity, whereas measuring CE-CT HU is a simple, easy method that does not depend on the physician’s skill.

In a voxel with average linear attenuation coefficient μ_*x*_, the corresponding HU value is given by: *HU* = 1000 × (μ_*x*_ - μ_water_)/μ_water_ - μ_air_ where μ_water_ and μ_air_ are the linear attenuation coefficients of water and air; i.e., attenuation values expressed in HU are relative to the attenuation of radiation in water. Positive values represent tissues with attenuation values higher than that of water and negative values represent tissues with lower values. The number 1000, sometimes called the magnifying value, is incorporated into the above equation to expand the scale sufficiently to provide whole number attenuation values [27]. Mean HU can be influenced by ROI selection, whereas maximal HU does not vary with ROI placement. Although selecting an ROI cannot currently be automated or standardized, it is an easy manual skill that requires no special technique or software. However, neighboring structures (e.g., bone or blood vessels) should be carefully avoided in selecting a ROI, as maximum HU markedly changes when structures other than lymph nodes are included. Further study is needed to clarify the accuracy of segmentation for ROI delineation; such investigation might show how much of a learning curve exists to acquire the stable ability to determine the HU value by non-experts. We found sensitivity, specificity, PPV, NPV, and accuracy were 79.6%, 80.5%, 70.9%, 86.8%, and 80.2%, respectively, at a cut-off value of 54 for maximum NC-CT HU. As this result was superior to the findings of previous studies [11, 17, 25], clinical use of this cut-off value is feasible. Furthermore, accuracy rates were 76.3%, 71.8%, and 68.7% for maximum CE-CT HU, mean NC-CT HU, and mean CE-CT HU, respectively. Therefore, the maximum NC-CT HU cutoff appears adequate to estimate ALN metastasis. Our results showed that NC-CT effectively detected metastasis, and is clearly superior to CE-CT and PET-CT, even from the viewpoint of side effects and cost. In any case, we recommend that HU be measured to evaluate lymph node metastasis when deciding preoperative staging.

The testing set results under blind conditions validated the analysis. The cut-off value for maximum NC-CT HU showed sensitivity, specificity, PPV, NPV, and accuracy of 100.0%, 76.7%, 58.8%, 100.0%, and 82.5%, respectively, which is particularly useful in light of how easily obtainable this measurement is in routine preoperative examinations.

To our knowledge, this is the first study to show that metastatic and negative ALNs differ in their mean and maximum HU values. Metastases that contain tumor cells, vascularization, or immune reactions within lymph nodes may elevate HU values. Although we did not evaluate the relationship between metastatic area and HU values, further studies should be performed to clarify this issue.

This study had three limitations. First, maximum HU may measure artifacts [28], and no conclusive evidence currently shows that maximum HU can accurately assess ALN metastasis. We also examined HU in 3-mm CT slices in this study; however, HU values on image borders may be inaccurate for small structures such as lymph nodes due to the partial volume effect. Secondly, HU values depend on the CT machine, imaging conditions, and specifications of the image processing software, which differ in every institution, and may vary due to maintenance or an update. A cut-off value for HU in detecting ALN metastasis may only apply to selected patients who undergo NC-CT screening in the same institution. Thus, cut-off values should be regularly recalibrated before treating patients. Finally, comparing one-to-one correspondences between resected lymph nodes and CT ALN images in the present retrospective study was difficult. Confirming relationships between preoperative CT HU and intraoperative and postoperative histological evaluations should resolve these problems. We will be able to achieve higher accuracy by measuring several ALNs followed by the selection of one node with the highest HU value.

## Conclusions

In conclusion, we have shown that measuring the maximum HU is a simple, easy, and useful technique for diagnosing ALN metastasis in breast cancer patients. However, as this was a retrospective study with relatively few patients, our results should be verified by a blinded prospective investigation by several researchers and a larger cohort.

## Abbreviations

ALN: Axillary lymph node
CE-CT: Contrast-enhanced computed tomography
CT: Computed tomography
FDG: ^18^ F-fluorodeoxy glucose
HU: Hounsfield unit
IDC: Invasive ductal carcinoma
NC-CT: Non-contrasting computed tomography
NPV: Negative predictive value
PET: Positron emission tomography
PPV: Positive predictive value
ROC: Receiver operating characteristic
ROI: Range of interest
SLN: Sentinel lymph node.
